# Mechanical Behavior of Masonry Mortars Reinforced with Disposable Face Mask Strips

**DOI:** 10.3390/ma17225571

**Published:** 2024-11-14

**Authors:** René Sebastián Mora-Ortiz, Ebelia Del Angel-Meraz, Sergio Alberto Díaz, Francisco Magaña-Hernández, Jazmín del Rosario Torres-Hernández, Mayra Agustina Pantoja Castro

**Affiliations:** División Académica de Ingeniería y Arquitectura (DAIA), Universidad Juárez Autónoma de Tabasco, Carretera Cunduacán-Jalpa de Méndez km. 1, Cunduacán 86690, Tabasco, Mexico; rene.mora@ujat.mx (R.S.M.-O.); alberto.diaz@ujat.mx (S.A.D.); francisco.magana@ujat.mx (F.M.-H.); jazmin.torres@ujat.mx (J.d.R.T.-H.); mayra.pantoja@ujat.mx (M.A.P.C.)

**Keywords:** facial mask, circular economy, waste recycling, fiber-reinforced mortar, mechanical properties

## Abstract

This research presents an experimental analysis of the mechanical behavior of masonry mortars incorporating disposable face masks (FMs) cut into two different sizes. The objective is to provide experimental data contributing to the consolidation of recycling FMs in mortar mixtures. To achieve this, two types of mixtures were prepared: one with strips of 3 × 3 mm and another with strips of 3 × 10 mm. These FM strips were added in different proportions by the volume of mortar (0%, 0.2%, 0.5%, 0.8%, 1.0%, and 1.5%). In all mortars, the dry bulk density, volume of permeable voids, and water absorption, as well as compressive, flexural, and tensile strengths, were evaluated after a 28-day water immersion curing period. Additionally, two essential properties in masonry mortars were analyzed: air content and shear bond strength. The results indicated that, for both strip sizes, adding FMs up to 0.2% positively affected the flexural and tensile strengths; concerning control mortar, increases of 6% and 1.4%, were recorded, respectively, for the longer strips. At this percentage, the density, air content, and compressive and shear bond strengths are not significantly affected. The results demonstrated that incorporating FMs into mortar mixtures is a promising avenue for sustainable recycling and helps reduce microplastic environmental contamination.

## 1. Introduction

The COVID-19 pandemic has led to massive use of disposable face masks as an essential protective measure against the spread of the virus. This increase in use has resulted in a significant accumulation of plastic waste, creating environmental and waste management challenges. Approximately 129 billion masks are estimated to be discarded worldwide each month, highlighting the urgency of developing effective recycling strategies to mitigate their environmental impact [1]. Disposable masks are primarily composed of polymers such as polypropylene (PP), which is difficult to degrade under natural conditions due to the strength of its structure, molecular weight, and hydrophobic properties [2,3]. As a result, a large amount of polypropylene in the form of microplastics accumulates in nature. This accumulation poses risks to wildlife and aquatic ecosystems, where marine organisms can ingest plastic particles, affecting their health and, potentially, human health [4,5].

Recycling disposable face masks (FMs) offers a potential solution to reduce waste volume and extend the lifecycle of valuable materials such as PP. Among various alternatives, the potential of incorporating recycled mask materials and other plastics into construction applications has recently been evaluated [6,7,8]. In this context, some researchers have addressed the reuse of facial masks in the form of fibers or shredded in the preparation of concrete and mortar mixtures [9,10,11]. As this is a new area of study, only a few studies have presented experimental analyses of the mechanical behavior of the mixtures. For example, in concrete preparation, Kilmartin-Lynch et al. [12] used FMs cut into pieces (2 × 0.5 cm) and added to cement pastes at different volume percentages. Their findings concluded that adding FMs up to 0.20% improves strength properties (compressive and flexural). Meanwhile, Koniorczyk et al. [13] prepared concrete mixtures with PP fibers (0.5 cm long and 0.5 mm in diameter) obtained from personal face masks. The experimental results showed that concrete with these fibers (at a proportion of one mask per liter of concrete) developed a 5% increase in compressive strength and a 3% decrease in tensile strength without affecting durability. Other researchers have reported that PP fibers obtained from FMs added up to 1% (by mixture volume) improve flexural strength and reduce shrinkage cracks in 3D concrete printing [14]. Yang et al. [15] and Ali et al. [16] have reported that adding FMs up to 1.5% and 1.0% by mixture volume, respectively, is beneficial for concrete properties. The authors caution that exceeding these percentages results in structural defects in the concrete, reducing compressive and tensile strength. All the studies above have reported that as the percentage of FMs increases, the workability of the mixtures decreases.

Recycling FMs into masonry mortar mixtures has attracted researchers’ interest recently. Ajam et al. [17] used FMs cut into pieces of 2 and 4 cm^2^, added in percentages of 1% to 5% by volume of mortar. They reported an improvement in acoustic and thermal resistance properties and an increase in compressive and flexural strength of up to 20% compared to the control mortar. Miah et al. [11] demonstrated that compressive strength decreases as the amount of shredded and cut FM increases while flexural strength increases. Additionally, they detected a decrease in shrinkage and water absorption for mixtures with FM percentages less than 1.5%. Similar results were published by Thwe Win et al. [18]. These authors reported that adding FMs in fiber form (especially 5 × 10 mm) increases flexural and tensile strength while decreasing compressive strength; however, it was noted that increasing the amount of FM fiber leads to a continuous increase in water absorption. The experimental results allowed them to conclude that the optimal percentage of FMs in mortar mixtures is 0.15%. Nie et al. [19] analyzed changes in the dynamic mechanical properties of mortars with shredded face masks added in different proportions by mixture volume. P-wave velocity tests showed that mortar integrity and fragmentation after impact improved with the inclusion of FM, with this improvement attributed to the reduction of microcracks. However, dynamic uniaxial compression strength decreased in all mortars with FM. The authors concluded that while improvements are observed in certain aspects of mechanical behavior, the incorporation of FM introduces internal defects and weakens the mortar structure.

Recycling FMs in masonry mortar production is a new and promising area. Compared to concrete, masonry mortar’s lower structural requirements make it one of the best options for recycling face masks. The PP present in FMs is of good quality and has high resistance to alkaline environments [15,20], making it an excellent material to add to mortar mixtures. However, much research is still needed regarding its effects on mechanical behavior and durability.

This research presents an experimental analysis of masonry mortars’ mechanical characteristics, incorporating disposable face mask strips cut into two different sizes. In addition to resistance properties, density, water absorption, and volume of permeable voids, two essential properties not previously directly addressed are analyzed: air content and shear bond strength. This paper aims to provide experimental data that contribute to characterizing the mechanical behavior of mortars with added FMs. It aims to broaden the scope of FM recycling in masonry mortar mixtures and help consolidate its reuse as an eco-friendly solution.

## 2. Materials and Methods

**Figure 1:**
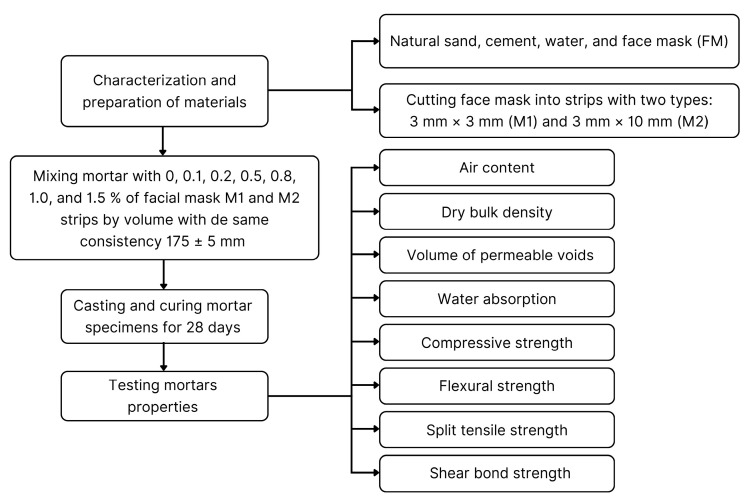
Flowchart of investigation.

Figure 1 shows the flowchart of this research’s experimental process. The proposed experimental program covered the basic tests for characterizing the mechanical behavior of masonry mortars.

### 2.1. Disposable Face Mask

**Figure 2:**
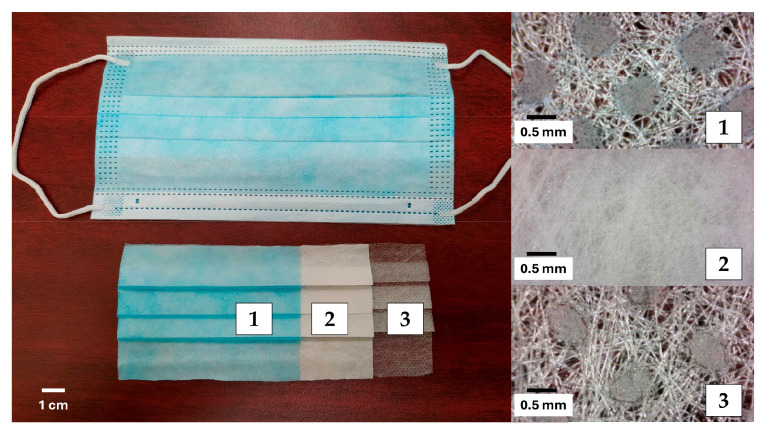
Layers of the disposable face mask.

To avoid infection risks and follow the university laboratory health regulations, only new disposable face masks of the same brand were used in this research [9,14,21]. Due to their widespread use and low cost, FMs consisting of three layers were utilized (Figure 2). These types of face masks are primarily made of polypropylene, with the outer layers made of non-woven fabric and the middle layer made of melt-blown fabric [22]. The structures of these layers are different because each serves a different function [17]. Figure 2 highlights that due to the arrangement of PP fibers, the internal structure of the FM layers contains voids and high porosity [9,23].

**Figure 3:**
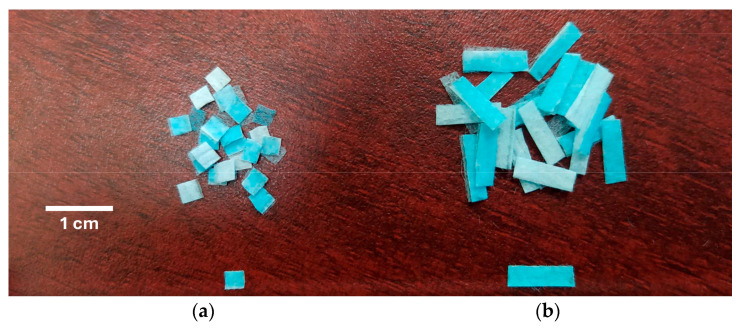
Fibers obtained from FMs: (**a**) type M1 and (**b**) type M2.

To incorporate the FMs into the mortar mixtures, the plastic supports located at the nose and ear areas were first removed, and then they were manually cut [16,20,21] into strips of two dimensions: 3 × 3 mm and 3 × 10 mm; these two types of strips were designated as M1 and M2, respectively (Figure 3). The thickness of the FMs was 0.71 ± 0.02 mm, with a specific gravity of 0.90 ± 0.02 g/cm^3^ and water absorption of 6.23 ± 0.12%. These properties were measured according to the ASTM D792 [24] and ASTM D570 [25].

### 2.2. Cement and Fine Aggregate

**Figure 4:**
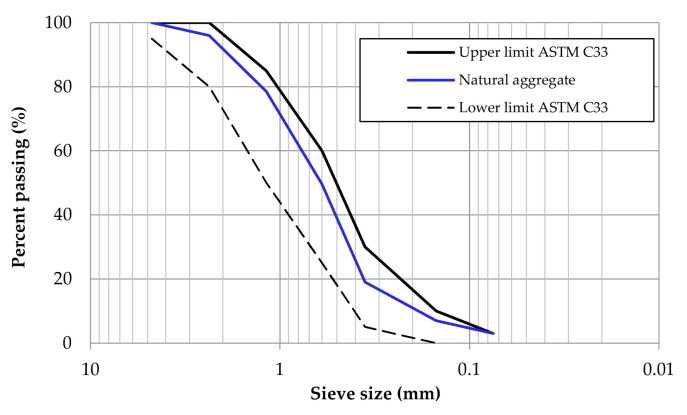
Particle size distribution of fine aggregate.

Ordinary Portland cement (CPO 30R) was used in the preparation of mortar mixtures, meeting the standards of ASTM C150 [26] and NMX-C-414-ONNCCE [27]. As natural aggregate (NA), river sand was used, with its particle size distribution determined according to ASTM C33 [28] and shown in Figure 4. Basic characterization tests of the NA were performed following ASTM C136 [29] and ASTM C128 [30]. The properties were as follows: fine content = 4.5%, specific gravity = 2.65 g/cm^3^, and water absorption = 1.25%.

### 2.3. Mix Proportions, Casting, and Curing

The composition used in the mortar mixtures of this research is shown in Table 1. The quantities of natural aggregate, cement, and mixing water are the same for all mixtures. Two families of mortars were designed, one for each type of FM strip (M1 and M2). The fibers were added to the mixtures in percentages of 0.1%, 0.2%, 0.5%, 0.8%, 1.0%, and 1.5% by volume of mortar. Thus, for example, the mix labeled M1-0.1 represents a mix supplemented with 0.1% of M1-type strips (3 × 3 mm). On the other hand, the M2-0.5 mix is supplemented with 0.5% of M2-type strips (3 × 10 mm). The total amount of water in the mixes was experimentally adjusted to achieve the target consistency of 175 ± 5 mm, which was determined according to ASTM C1437 [31]. A 10-inch mechanical flow table from ELE International (Milton Keynes, UK) was used.

The mix preparation was based on the ASTM C305 procedure [32]. First, the water and FM strips were placed in the mixer bowl (for those mixes containing facial masks). Then, the cement was added, and these components were mixed at low speed (140 rpm) for 30 s. Next, all the sand was gradually added over 30 s while the mixer continued operating. Once all the elements were added, mixing continued for 1.5 min. After this time, the mixer was stopped, and the mix was manually stirred for 30 s. Finally, the mixture was re-mixed in the mixer at low speed for another 1.5 min. An electrically driven, three-speed, epicyclic-type mechanical mixer from Hobart (Offenburg, Germany) was used.

The fresh mixes were placed in their respective molds and allowed to set. After 24 h, the specimens were removed from the molds and subjected to a curing process by immersion in water for 28 days. All activities were conducted in a laboratory with a controlled temperature of 23 ± 2 °C and a relative humidity of 50%.

### 2.4. Rehearsal Program

In all the tests described below, the final result reported was the arithmetic mean of at least three samples for each type of mix.

The air content was determined [33] immediately after the mix was prepared using the procedure in ASTM C185 [33]. It was calculated using Equation (1) based on the mortar densities, the known densities of the components, and the mix proportions.

(1) $$Air\;content,\;volume\;\%=100-W\left[\left(182.7+P)/(2000+4P\right)\right]$$

where W is the mass of 400 mL of mortar, and P is the percentage of mixed water relative to the cement mass.

The dry bulk density, volume of permeable voids, and water absorption of the mortars were obtained using 50 mm cubic samples and following the procedures established by ASTM C642 [34]. These properties were calculated using Equations (2)–(4), respectively, by measuring the masses in different states: oven-dry, saturated mass after immersion, saturated mass after boiling, and immersed apparent mass.

(2) $$Dry\;bulk\;density=\left[A/(C-D)\right]\cdot \rho$$

(3) $$Volume\;of\;permeable\;voids=\left[\left(C-A)/(C-D\right)\right]\times 100$$

(4) $$Absorption=\left[(C-A)/A\right]\times 100$$

where A denotes the mass of the oven-dried sample in air, C is the mass of the surface-dry sample in air after immersion and boiling, D is the apparent mass of the sample in water after immersion and boiling, and ρ is the density of water.

The compressive strength was obtained according to the procedure described in ASTM C109 [35] using 50 mm cubic samples. The flexural strength was determined using prismatic samples of 40 × 40 × 160 mm, tested under a central load as established in ASTM C348 [36]. The loads were applied using a universal testing machine from DAVI (Estado de Mexico, Mexico) with a maximum load capacity of 120 tons.

The splitting tensile strength test was conducted on 100 × 200 mm cylindrical specimens according to ASTM C496 [37]. This test applies a diametral tensile force along the length of the specimen until failure occurs. The splitting tensile strength was determined using Equation (5):

(5) Split tensile strength=2P/πld

where P, l, and d are the specimen’s maximum applied load, length, and diameter.

**Figure 5:**
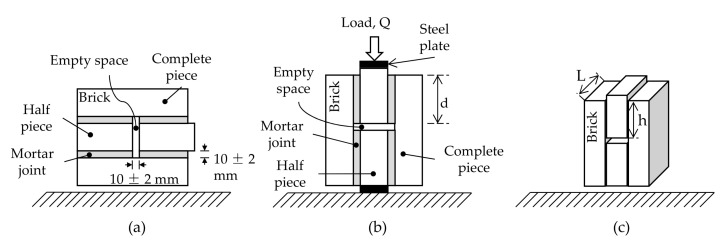
Shear bond strength specimen: (**a**) manufacturing position; (**b**) testing position; (**c**) dimensions of specimen.

The shear bond strength of the mortars was determined according to NMX-C-082-ONNCCE [38]. Five samples were made for each mix type. Each sample was composed of two 50 × 100 × 200 mm and two 50 × 100 × 100 mm pieces. These pieces were joined with the respective mix under analysis, as shown in Figure 5a.

After construction, the samples remained still for 28 days in a temperature-controlled (23 ± 2 °C) laboratory. They were tested using a universal testing machine (Figure 5b). According to the standard NMX-C-082-ONNCCE [38], the shear bond strength was computed using Equation (6):

(6) $$SBS=\frac{Q}{A}=\frac{Q}{2Lh}$$

where Q represents the load that induces failure, A is the total area where the load is applied, and L and h are the thickness and height of the brick-and-mortar-covered surface.

## 3. Results and Discussion

### 3.1. Air Content

**Figure 6:**
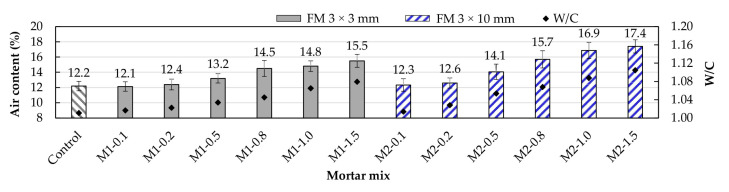
Changes in air content due to the inclusion of FM.

Figure 6 shows the air content test results and the variations in the mortars’ water–cement (*w*/*c*) ratio. It was noted that the air content also increased as the amount of FM increased. This is due to three reasons: first, the hydrophobic nature of the FM strip surface caused air void formation between the cement paste and the PP fiber surface [13,39,40], leading to the formation of an ITZ (interfacial transition zone) [41,42]. Second, as the number of strips gradually increased, they intertwined, creating voids and trapping air during mixing [43,44]. Third, adding FM to the mixes reduced their workability, so to achieve the same consistency (175 ± 5 mm), it was necessary to adjust the amount of water, increasing the *w*/*c* ratio (see Table 1). Researchers such as Wang et al. [45], Sayed et al. [46], and El-Newihy et al. [47] reported increases in the air content of concrete samples caused by an increase in the content of various types of fibers (including polypropylene).

Although both types of mortars showed the same trend, it was observed that the M2 mortars exhibited higher air content values compared to their M1 counterparts. For example, the M2-0.5, M2-0.8, M2-1.0, and M2-1.5 mortars showed increases of 6.8%, 8.3%, 14.2%, and 12.3%, respectively, relative to their M1-type counterparts. These results indicate that fiber length directly affects air content, with longer fibers combined with a progressive increase in the number of strips leading to excessive intertwining, causing an increase in ITZ, porosity, and air content [44].

### 3.2. Dry Bulk Density

**Figure 7:**
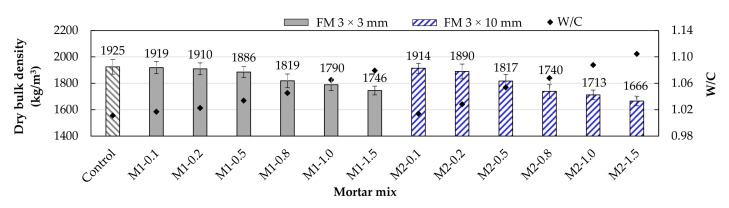
Dry bulk density of the mortars with different proportions of FM.

Figure 7 shows the changes in the hardened state density of the mortars due to the addition of FM. The results showed that the density decreased for both types of mortar as FM was added to the mixes. This occurs due to the low density of the strips themselves and the increase in air content and *w*/*c* ratio associated with the inclusion of FM strips. These results align with those reported by other researchers in studies conducted with PP fibers [18,48].

As seen in Figure 7, the M2-type mortars exhibited the lowest densities compared to their M1-type counterparts. Note that for FM contents of 0.1% and 0.2%, the densities in both types of mortars were similar to that of the control mortar. As the FM content increased, the differences between the mortars grew. For example, for FM contents of 0.5%, 0.8%, 1.0%, and 1.5%, the M2-type mortars had densities 3.7%, 4.34%, 4.3%, and 4.6% lower than their M1-type counterparts. This suggests that including larger strips causes increased intertwining between them [46] as well as increased air content, directly reflected in the mortar density.

### 3.3. Volume of Permeable Voids

**Figure 8:**
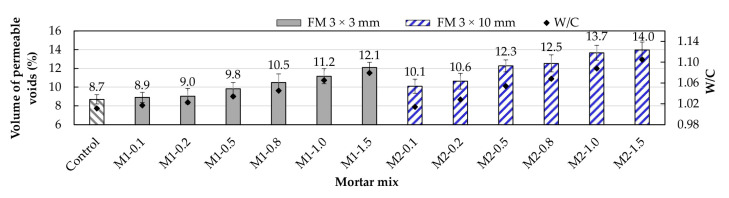
Volume of permeable voids of the mortars.

**Figure 9:**
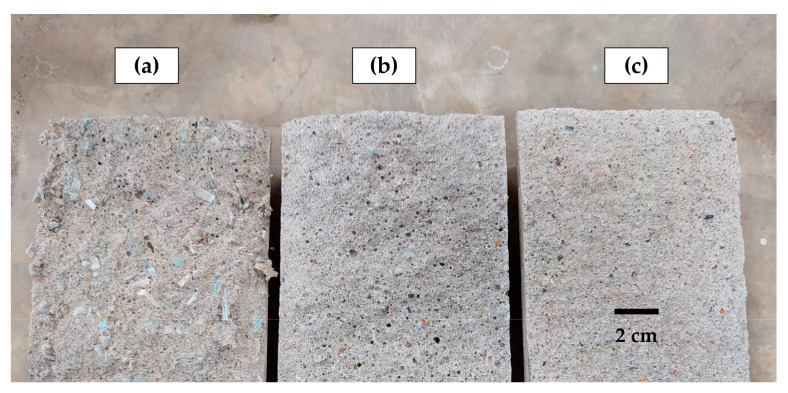
Porosity of samples M2-type with different FM content: (**a**) 1.5%; (**b**) 0.5%; (**c**) control.

As seen in Figure 8, for both types of mortar, the volume of permeable voids increased as the amount of FM strips increased. This trend occurred due to the progressive increase in air content in the mortar mixes (Section 3.1). Figure 9 shows the comparison of the porosity levels in mortars with varying FM contents. These results align with the observations of researchers such as Islam et al. [49] and Karahan and Atis [50], who reported a growing increase in the porosity of concrete samples due to the progressive inclusion of PP fibers, resulting in increased permeability to water and gas. In a study conducted on concrete samples, Rajeev et al. [14] concluded that when the fiber content from FMs increases, the viscosity and yield strength also increase, reducing fluidity and increasing the likelihood of pore formation.

M1-type mortars exhibited fewer pores compared to their M2-type counterparts. Figure 8 shows that for FM contents of 0.5%, 0.8%, 1.0%, and 1.5%, M1-type mortars had 25.5%, 19.0%, 22.3%, and 15.7% less pore volume than their M2-type counterparts. This again highlights that longer strips negatively impact mortar properties [18]. It can be noted that within M1-type mortars, those with 0.1% and 0.2% showed porosity close to the reference mortar value (8.7%), indicating that for these percentages, the fibers were better distributed in the matrix, reducing the possibility of void formation.

Thus, for FM contents greater than 0.2%, porosity increases rapidly with the strip content, which is caused by air trapped both between the cement paste and the strips themselves, as well as due to the interlocking between them [9]. This effect is more pronounced in M2-type mortars due to the greater length of their strips (10 mm). Figure 9a shows an example of the excessive interlocking of FM strips in an M2-1.5 mortar sample. Figure 9b,c show the porosity of the M2-0.5 mortar and the control mortar, respectively, demonstrating that as the FM strip volume decreases, porosity also decreases.

### 3.4. Water Absorption

**Figure 10:**
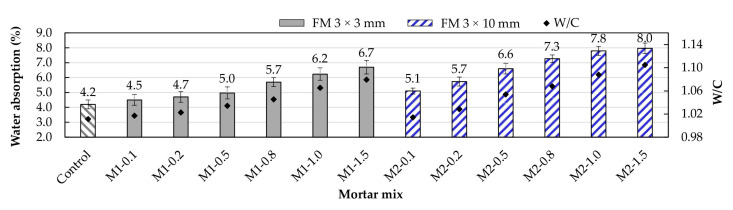
Changes in the water absorption of the mortars due to the inclusion of FM.

Water absorption is an important parameter as it indicates the compactness and durability of mortar and concrete samples [51,52]. The results of the absorption test conducted in this study are shown in Figure 10. In both types of mortar, water absorption increased with the FM content. The air voids in the ITZs (interface between strips and cement paste) and the interlocking of the strips caused an increase in porosity, which directly impacted water absorption [18]. It can be observed that M2-type mortars showed higher absorption due to their greater porosity (Section 3.3). Furthermore, Ajam et al. [17] and Islam et al. [49] noted that the strips act as micropore bridges between the pores, facilitating intercommunication and, thus, leading to more significant water migration. Comparing water absorption in the mortars for FM contents of 0.5, 0.8, 1.0, and 1.5%, it is evident that M2 mortars absorb 19 to 32% more water than M1 mortars, highlighting the negative effect of longer strips.

In contrast, M1-type mortars with FM percentages of up to 0.2% showed water absorption levels similar to that of the control mortar. This suggests that for smaller fiber sizes and contents below 0.2%, the strip dispersion is uniform [53], the number of pores developed does not significantly affect water absorption, and the water migration effect due to micropore bridging is reduced.

### 3.5. Compressive Strength

**Figure 11:**
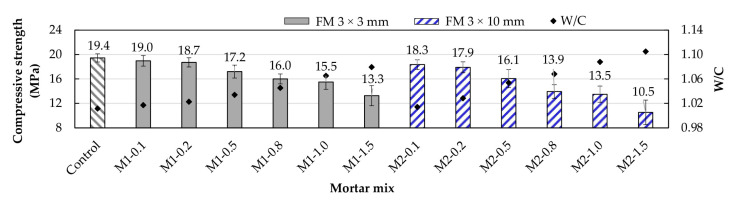
Compressive strength of mortars with different FM contents.

The compressive strength test results for the control samples and the mortars supplemented with FM are presented in Figure 11. Compared to the control mortar, all strengths decreased with an increase in FM volume. This trend was more pronounced in M2-type mortars. Comparing M2-0.5, M2-0.8, M2-1.0, and M2-1.5 mortars with their M1 counterparts, it was observed that M2-type mortars are 6.4%, 13.1%, 12.9%, and 21.1% less resistant, respectively. This behavior is due not only to the inherent increase in porosity from adding FM to the mixes but also to redistributing the strips. As the volume increases, their interlocking intensifies, creating weak zones where the ITZs are more developed [9,49,50,54]. Idrees et al. [44], Thwe Win et al. [18], and Xie et al. [43] concluded that increasing fiber content (including fibers obtained from recycled FMs) results in increased air content in the mixes, which in turn decreases density and, finally, compressive strength.

In both types of mortars (M1 and M2), it was observed that for FM percentages up to 0.2%, the decrease in strength is not as pronounced as for higher percentages, with M1-type samples showing strength levels very similar to the control mortar. This indicates that the adverse effects on compressive strength are not significant for FM percentages up to 0.2% and shorter strip lengths. Yoo et al. [55] conducted a study on concrete samples supplemented with fibers of different lengths, concluding that shorter fibers show better orientation and dispersion, improving strength properties. Similarly, Koniorczyk et al. [13] reported a 5% increase in the compressive strength of concrete samples supplemented with short fibers (5 mm in length) obtained from the thermal processing of FMs.

### 3.6. Flexural Strength

**Figure 12:**
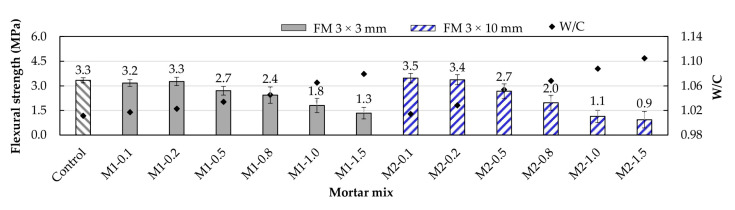
Flexural strength of the mortars under study.

Figure 12 displays the variations in the flexural strength of the mortars under study. Compared to the control mortar’s flexural strength, it was observed that for the M1-type mortars with 0.1 and 0.2% FM, the strengths were similar, while for the same FM contents in M2-type mortars (M2-0.1 and M2-0.2), an increase of 6.1% and 3.0%, respectively, was recorded. The results indicate that the volume of pores is reduced for low FM contents, and the strip distribution in the mortar is adequate to minimize interlocking. Anas et al. [56], Shen et al. [57], and Nie et al. [19] mentioned that in the absence of excessive interlocking, reinforcement fibers act as micropore bridges or anchors between tension zones within the mortar, better distributing the loads and intercepting microcracks.

M2-type mortars showed greater flexural strength for 0.1 and 0.2% FM contents because of their greater length, which allowed for better load distribution. These results are consistent with what various researchers have reported regarding the use of PP fibers as reinforcement in concrete and mortar mixes [18,55,58]. Aziz et al. [21] concluded that longer fibers help achieve greater ductility in concrete samples and improve the connection between a single FM fiber and the elements of the mix.

In both types of mortars, it was observed that flexural strength decreases for FM percentages above 0.2% as the FM content increases. This is due to increased porosity, poor strip distribution, and greater interlocking between them [17,59]. Rajeev et al. [14] concluded that for 3D-printed concrete, 1% fiber content of FM is sufficient to ensure correct distribution, increasing flexural strength and reducing crack formation. Koniorczyk et al. [13] also concluded that the benefit of using FM fibers in concrete samples depends on the effective dispersion of the fibers.

### 3.7. Split Tensile Strength

**Figure 13:**
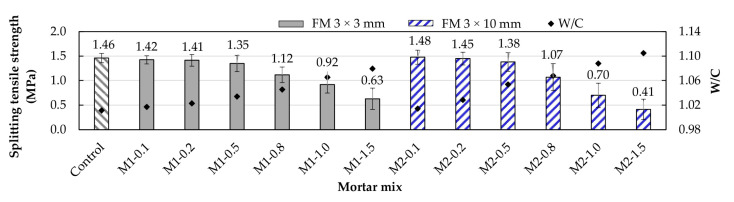
Split tensile strength of mortars with different FM contents.

**Figure 14:**
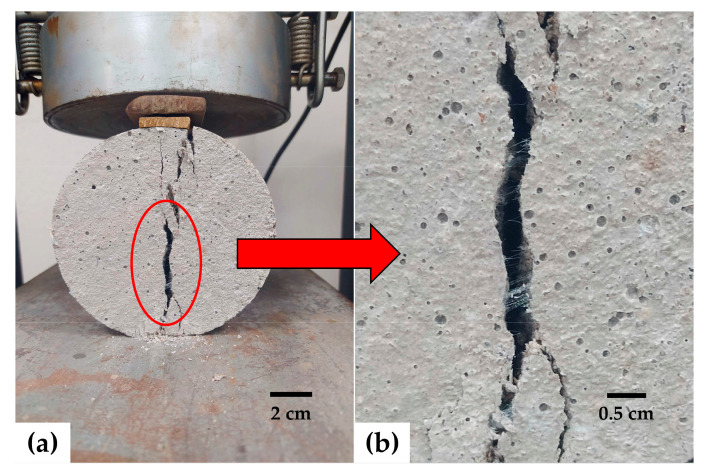
Split tensile strength test: (**a**) failure of the M2-0.5 sample; (**b**) micro-anchor effect.

The split tensile strength test results are shown in Figure 13. In both types of mortars, the highest strengths were observed for FM contents of 0.1 and 0.2%. As mentioned in the case of flexural strength, this is due to the strips acting as micro-anchors, reinforcing areas under tension (Figure 14). In low FM percentages, the volume of pores is reduced, the strip distribution is better, and clumping is minimized. Ajam et al. [17] and Al-Hadithi et al. [60] agree with this and added that a good distribution of strips requires more energy to propagate microcracks, resulting in increased strength. Similarly, Ahmed et al. [9] and Kilmartin-Linch et al. [12] reported that for the same range of PP fiber contents (0.1 and 0.2%) obtained from FMs, strength increases of up to 12% were achieved in concrete samples. Figure 13 shows that when FM contents of 0.5% and above were reached in both types of mortar, the trend was that greater FM volume resulted in lower strength. This is consistent with the findings of the flexural strength test.

Comparing M2-type mortars with their M1 counterparts, it was observed that for FM contents of 0.1%, 0.2%, and 0.5%, M2 mortars exhibited strengths of 4.2%, 2.8%, and 2.2% higher, respectively. This is due to the longer fibers in M2 mortars, which, as mentioned in previous sections, better distribute the loads. For FM contents greater than 0.5%, it was observed that M1-type mortars showed higher strength than M2 mortars. This is because, at higher quantities, the fiber dispersion is not correct, and longer fibers tend to interlock more [18,59].

### 3.8. Shear Bond Strength (SBS)

**Figure 15:**
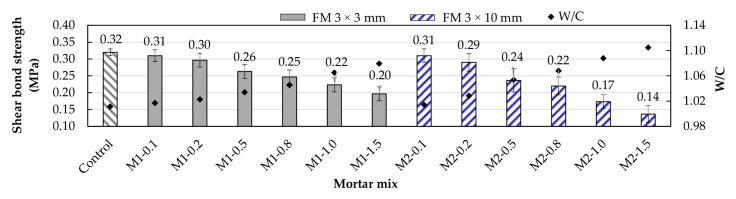
Changes in shear bond strength due to the inclusion of FM.

**Figure 16:**
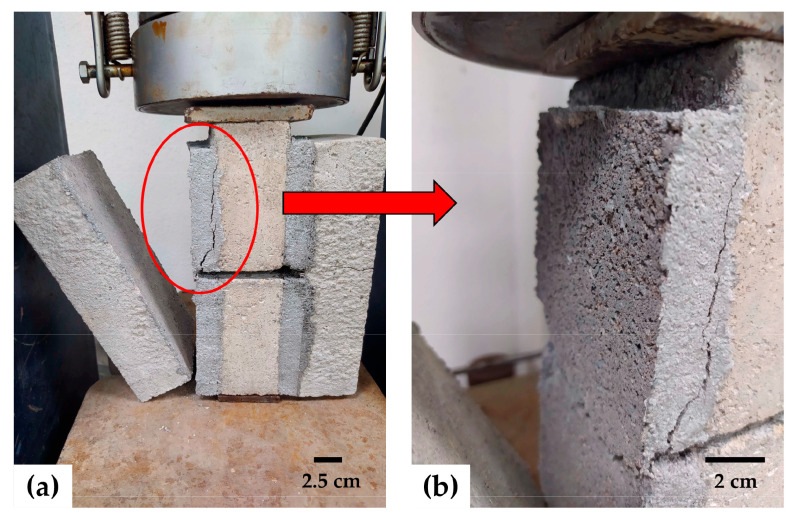
M2-1.0 mortar after the SBS test: (**a**) adhesive failure; (**b**) porosity in the mortar paste at the mortar–base material interface.

Shear bond strength refers to the ability of mortar to withstand shear forces applied along an interface between the mortar itself and the material it is bonded to. Figure 15 shows the SBS values of the mortars under study. In both types of mortar, SBS decreased as the FM content increased. The mortars with the highest shear bond strengths were those with strip contents of 0.1 and 0.2%, although they were still below the control value. After 0.2% FM content, SBS decreased significantly with increasing FM. In all cases, an adhesive-type failure occurred at the interface between the mortar and the base material (brick or block). Figure 16 shows this type of failure in an M2-1.0 sample.

The general decrease in sample strength is mainly due to the increased air content associated with including FM (Section 3.1). A higher air content can hinder the adhesion of the mortar to the base material, as air bubbles interrupt direct contact between the mortar and the brick or block, reducing the effective bonding area and, in turn, lowering the shear bond strength of the joint. Additionally, the increase in air content makes the mortar less dense (Section 3.2), which reduces the amount of solid material per unit volume. This implies less direct physical contact between the mortar and the base material, further reducing adhesion at the interface. Furthermore, due to the nature of the surface of FM fibers, they do not adhere well to other surfaces on their own, so their inclusion in mortar mixes does not contribute to increasing adhesion. On the contrary, when the fibers in the mortar come into contact with the surface to which the mortar is to adhere, the effective bonding area is reduced. Karahan et al. [50] demonstrated that the redistribution of pores due to the inclusion of PP fibers generates weak interfacial bonds between the fibers and the cement paste. Ajam et al. [17] also mentioned that when the three layers of FM fibers remain intact during mixing, the cement paste cannot penetrate them, resulting in reduced adhesion and significant porosity.

## 4. Conclusions

This study presented an experimental analysis of the mechanical behavior of masonry mortars supplemented with disposable facial masks (FM). The goal was to provide experimental data to help expand and consolidate the recycling of these materials. The results of the laboratory tests led to the following conclusions:

- Because FM strips are hydrophobic, including them in mortar mixes increases the air content. The larger the strip size, the higher the air content.
- Overall, the progressive inclusion of FM strips in the mortar mixes reduced the mechanical properties analyzed.
- The FM contents that performed best in both types of mortars were 0.1% and 0.2%. For these amounts, the better dispersion of strips in the mixes ensured that the included air and permeable voids remained close to control mortar values. Hence, the other analyzed properties were not significantly affected.
- M1-type mortars, with shorter strips, exhibited better distribution than larger strips (M2) and, generally, showed less air inclusion. As a result, M1-type mortars showed better values for water absorption, volume of permeable voids, and dry bulk density, as well as compressive and shear bond strengths.
- For FM contents of 0.1% and 0.2%, the longer strips (M2) demonstrated better flexural and split tensile strengths due to the micro-anchor effect and their better load distribution.

The sustainable recycling of FM in masonry mortar mixes is feasible up to 0.2% by volume. The use of air-reducing additives can significantly improve mortars’ mechanical performance. These results are promising; however, further studies on durability are also needed. Recycling FMs helps reduce environmental pollution by microplastics and increases the lifecycle of these materials.

## Figures and Tables

**Table 1:** Mortar mix proportions.

Mortar Mix	Facial Mask Type	NA (g)	CEM (g)	Mixing Water (g)	Total Water (g)	Facial Mask (%)	Consistency Index (mm)	W/C
Control		1573	353	357	357	0	174	1.011
M1-0.1	M1 3 × 3 mm	1573	353	357	359	0.10	175	1.017
M1-0.2		1573	353	357	361	0.20	171	1.023
M1-0.5		1573	353	357	365	0.50	178	1.034
M1-0.8		1573	353	357	369	0.80	170	1.045
M1-1.0		1573	353	357	376	1.00	176	1.065
M1-1.5		1573	353	357	381	1.50	179	1.079
M2-0.1	M2 3 × 10 mm	1573	353	357	358	0.10	170	1.014
M2-0.2		1573	353	357	363	0.20	173	1.028
M2-0.5		1573	353	357	372	0.50	175	1.054
M2-0.8		1573	353	357	377	0.80	176	1.068
M2-1.0		1573	353	357	384	1.00	174	1.088
M2-1.5		1573	353	357	390	1.50	177	1.105

## Data Availability

Data are contained within the article.
